# Mercy Hospital

**Published:** 1880-04

**Authors:** E. C. Helm


### Clinical Reports.

---

### NOTES FROM HOSPITAL PRACTICE.

### ARTICLE V.

MERCY HOSPITAL. IN THE SERVICE OF PROF. N. S. AND DR. F. H. DAVIS. (REPORTED BY E. C. HELM, M.D.)

*Typhoid Fever.*

Fully one-half of the cases of typhoid fever treated in Mercy Hospital during the past few years have had no diarrhœa, some being constipated during the entire course of the disease and others having one natural daily movement of the bowels.

Of the cases of diarrhœa, those in which there was copious hæmorrhage from the bowels during the second or third week were regarded as being in the greatest danger, particularly if the tongue became very dry and brown, with sordes on the teeth, low muttering delirium, subsultus tendinum, and sudamina on neck and chest.

The last mentioned symptoms being regarded as almost surely indicating speedy collapse.

The treatment has been: 1. Hygienic. 2. To prevent deterioration of the blood. 3. To restore the natural secretions. 4. To reduce the temperature. 5. To control the action of the bowels. 6. To maintain innervation. 7. To control the action of the heart.

The air is kept pure and wholesome, at as nearly as possible a uniform temperature of 65°—70° F.

The bedding and underclothing are kept clean—are aired and changed frequently.

All excretions are at once removed from the ward.

The patient is kept clean, cool, and as comfortable as possible, by frequent spongings with tepid water, to which is added salt, or preferably vinegar, thereby partially controling the temperature, favoring diaphoresis and greatly adding to the comfort of the patient.

They are fed with the blandest nourishment, such as milk, thin wheat or oat-meal porridge, beef tea, etc., but the main reliance is placed upon the milk, beaten up with the white of an egg and sugar. From (ʒi–ij) four to eight grams of this are given four or five times a day during the first week, after which smaller quantities, from (ʒss–i) two to four grams, are given every hour, as starvation is believed to have a prominent place in the causes of death from typhoid fever.

When thirsty, the patients are given small pellets of ice to hold in the mouth; milk-whey, or better still, buttermilk, is often given, thus forming a cool, pleasant, yet diuretic drink.

For the first day or two potas. chlorate, (gr. iii) .250 gms. and acid hydrochloric, (grs. ij–iv) .130 to .250 gms. every three hours, alternating, if the bowels are constipated with minute doses of calomel and ipecac. If, on the contrary, the bowels are loose, pulv. dov. is used.

After the first thirty-six hours:

| | | | *Gms.* |
|---|---|---|---|
| ℞ | Acid salicyl.......................... | (℥ss) | 15 |
| | Sod. bicarb.......................... | (ʒijss) | 10 |
| | Glycerinæ .......................... | (ʒx) | 40 |
| | Aquæ .......................... | (℥iv) | 120 |

A dessertspoonful of this is given every two to four hours, alternating with spongings.

This is continued until the temperature is reduced to 100° F. and then just enough of the salicylic acid is used to keep it there.

Should there be much diarrhœa, enough tr. opium is added to check it.

Tr. digitalis is usually added to the above formula to act as a diuretic, but more especially to keep the heart within bounds. Digitalis has been found especially beneficial when the heart was very weak and feeble. Should the tr. digitalis be not sufficiently diuretic, liquor ammon. acet. or spts. æth. nit. is added.

During the second week, if the bowels are very loose:

| | | | Gms. |
|---|---|---|---|
| ℞ | Olei terebinth | (ʒiii) | 15 |
| | Tr. opii | (ʒv) | 20 |
| | Olei gaulth | (ʒss) | 2 |
| | Pulv. acaciæ | (℥i) | 30 |
| | Sacch. alb | (℥i) | 30 |
| | Aquæ | (℥iv) | 120 |

Of this (ʒi) four grams is given every four hours. This has usually promptly controlled the bowels, but in a few cases the turpentine has disagreed with the stomach or caused symptoms of strangury. In these cases it was promptly discontinued and a pill consisting of

| | | | Gms. |
|---|---|---|---|
| ℞ | Argent nit | (gr. ⅓) | 02 |
| | Ext. Hyoscyam | | |
| | Pulv. opii | (āā gr. j) āā | 06 |

substituted.

The nitrate of silver is not continued long, as permanent discoloration of the skin is feared, but after three to six days is replaced by

| | | | Gms. |
|---|---|---|---|
| ℞ | Acid sulph. arom., magnes. sulph | | |
| | Tr. opii | (ʒij) āā | 8 |
| | Syr. simp | | |
| | Aquæ | (℥i) āā | 30 |

Of this a teaspoonful is given every three to four hours while the diarrhœa lasts.

Should low, muttering delirium, subsultus-tendinum, petechiæ, etc. ensue, indicating nerve failure, the patients are given

| | | | Gms. |
|---|---|---|---|
| ℞ | Strychniæ sulph | (gr. $\frac{1}{32}$) | 002 |
| | Acid nitric | (gtts. iij) | 2 |

every two hours.

Brandy or any of the other so-called alcoholic stimulants are not given, as it is believed that they have a positively depressing effect, and in too many cases turn the scale of the patient's life toward a fatal end.

The diffusible stimulants, notably arom. spts. of ammon. or carbonate of ammon., are freely used and with decided benefit.

The results of the above treatment have been decidedly favorable, the death ratio being much below that given as the average, in hospitals where alcoholic remedies are freely used.

Large doses of quinine, or cold baths have been found to be efficient, yet dangerous, methods of reducing the temperature, the quinine often enfeebling innervation, while the shock and subsequent reaction following the use of cold baths, have more than counterbalanced its good effects.

During convalescence, if the patients are constipated, they are given

| | | Gms. |
|---|---|---|
| ℞ Ext. Hyoscyam | | |
| Ext. Leptand | | |
| Ferri. sulph. ext | (gr. i) āā | 06 |
| Ext. nux vom | (gr. ⅓) | 02 |

S. One at night.

If the bowels are loose they are given

| | | Gms. | |
|---|---|---|---|
| ℞ Strychniæ | (gr. i) | | 06 |
| Acid nitrici | (ʒ i) | 4 | |
| Tr. opii | (℥ss) | 16 | |
| Aq. and syr. q. s. ad | (℥ iv) | 120 | |

Mix. S. Teaspoonful three or four times a day.

Some of the bitter tonics, as tr. cinch. comp., are found of use. Special attention is paid to diet during convalescence, the food being plain, wholesome, nutritious, yet bland and unirritating. The patients are to avoid exposure to drafts of air, inclement weather or over exertion for several weeks.

